# Divorce Law

**Published:** 1920-03-20

**Authors:** 


### THE PUBLIC WELL-BEING.

### DIVORCE LAW.

It was an interesting discussion which took place in the House of Lords on Lord Buckmaster's Bill for the reform of the divorce law on the lines of the majority report of the Royal Commission on Divorce, 1912. The conclusion reached in this report was that unless the union formed by marriages which have already ceased in fact can be dissolved in law, lives become hopelessly miserable, illegal unions are formed, immorality results, and illegitimate children are born. Generally speaking, adultery is now the only legal ground for divorce, and Lord Buckmaster's Bill proposes to extend the ground to desertion for three years, without the consent and against the will of either party; cruelty, habitual insanity after five years, habitual drunkenness found incurable after three years of separation, and imprisonment under a commuted death sentence. It also provides for cheaper legal processes for divorce. These, of course, are big changes, and the actual details are subjects for discussion. But while many still respect the Church's attitude, as expressed by the Archbishop of York and Lord Phillimore, there are also many others who can see no common-sense, practical ground for refusing to reform the divorce law. In common justice it must at least be made accessible equally for the poor as for the rich. The reformers urge that if the State clings to the strict and narrow orthodoxy of the inviolate marriage tie, it must reckon with great laxity of morals and great injustice to children born out of wedlock. It is beyond dispute that thousands of married people are living permanently apart for some reason or other, and the numbers have been increased by the reckless emotionalism of many war marriages. What is to be gained, say the reformers, by insisting that the State should continue to penalise those who suffer from this unhappiness, and more especially illegitimate children? With proper safeguards and limitations it seems to these enthusiasts reasonable to regularise the existing state of things in the interests not only of happiness, but also of morality. No law will ever prevent deserted or wronged wives and husbands from seeking consolation elsewhere, and it is argued that they should have the chance of bringing to the Courts wider issues for consideration than those of adultery. It is true, no doubt, the hypocrisy of present divorce proceedings is patent. It was fearlessly exposed some time ago by Colonel Josiah Wedgwood, M.P.; and in Mr. Somerset Maugham's recent play. Collusion is over and over again obvious to the lay mind; the parties have only to go through certain "elaborate farces" in order to satisfy legal requirements, provided they can afford the price. The State should, at any rate, be honest with itself by facing the situation squarely, and by making the law coincide with realities. To do otherwise can surely appeal neither to the ecclesiastical nor to the reforming mind.

